# Linear Skeletal Muscle Index and Muscle Attenuation May Be New Prognostic Factors in Colorectal Carcinoma Treated by Radical Resection

**DOI:** 10.3389/fonc.2022.839899

**Published:** 2022-02-23

**Authors:** Yang Wang, Yuliuming Wang, Lianjie Ai, Hao Zhang, Guodong Li, Zitong Wang, Xia Jiang, Guoqing Yan, Yunxiao Liu, Chunlin Wang, Huan Xiong, Guiyu Wang, Ming Liu

**Affiliations:** ^1^ Department of Colorectal Surgery, The Second Affiliated Hospital of Harbin Medical University, Harbin, China; ^2^ Department of General Surgery, The Fourth Affiliated Hospital of Harbin Medical University, Harbin, China; ^3^ Department of Colorectal Surgery, Cancer Hospital of the University of Chinese Academy of Sciences/Zhejiang Cancer Hospital, Hangzhou, China

**Keywords:** colorectal carcinoma, linear skeletal muscle index, body composition parameters, tumor-related factors, prognostic factors, computed tomography.

## Abstract

**Objective:**

This study evaluated the association between body composition and clinical parameters and prognosis in patients with colorectal cancer (CRC) treated by radical resection.

**Methods:**

Baseline data on patient age, body mass index (BMI), bowel obstruction and tumor-related factors were collected retrospectively. Body composition parameters such as visceral fat area (VFA), total abdominal muscle area (TAMA), muscle attenuation (MA), posterior renal fat thickness (PPNF) and intermuscular fat area (IMF) are measured using Computed tomography (CT) scans. We also propose a new predictor of linear skeletal muscle index (LSMI) that can be easily measured clinically at CT. Follow-up endpoints were disease-free survival and all-cause death. We follow up with patients in hospital or by telephone. Univariate and multifactorial Cox proportional hazards analyses were performed to identify risk factors associated with prognosis. Survival analysis was performed using the Kaplan-Meier method and a nomogram was established to predict survival.

**Results:**

A total of 1761 patients (median age 62 years) with CRC were enrolled in our study, of whom 201 had intestinal obstruction and 673 had a BMI > 24.0. Among all patients, the 3- and 5-year disease-free survival rates were 84.55% and 68.60% respectively, and the overall survival rates were 88.87% and 76.38%. Overall survival was significantly correlated with MA, LSMI, SMI, Tumor size, N stage, metastasis and adjuvant therapy by Cox regression analysis (p < 0.05). The risk of tumor progression was significantly associated with MA, VFA, LSMI, SMI, Male, N stage, metastasis and adjuvant therapy (p < 0.05). In addition, based on the Chinese population, we found that female patients with MA < 30.0 HU, LSMI < 18.2, and SMI < 38.0 had a worse prognosis, male patients with MA < 37.6 HU, LSMI < 21.9, and SMI < 40.3 had a poorer prognosis.

**Conclusion:**

Our findings suggest that linear skeletal muscle index and MA can be used as new independent predictors for colorectal cancer patients treated with radical surgery, and that baseline data such as body composition parameters, LSMI and tumor-related factors can collectively predict patient prognosis. These results could help us to optimize the management and treatment of patients after surgery.

## Introduction

Colorectal cancer is the third most common cancer worldwide, with a total of 935,173 deaths from colorectal cancer in 2020 (1). In China, the incidence and mortality of colorectal cancer are increasing simultaneously, which is one of the worst among the three models proposed by Arnold et al. for the recent trend of colorectal cancer prevalence in the world (2).

Studies have reported that a higher BMI is associated with a series of endocrine and metabolic changes related to cancer development (3). However, in patients with gastric cancer (4) and colorectal cancer (5), a higher BMI at diagnosis is not associated with a higher risk of death, resulting in an obvious “obesity paradox”. BMI is an imprecise body composition. It cannot distinguish between muscle and adipose tissue, nor can it describe the different proportions of adipose tissue and lean tissue (6), and cannot represent obesity well, thus confusing the health consequences of morbidity and mortality (7–9). Recent observational studies have shown that adipose tissue and muscle distribution are risk factors for postoperative complications and overall survival in cancer patients (10, 11). Furthermore, body composition may be further aggravated by cancer and cancer treatment, highlighting the importance of body composition in oncology (12, 13).

Body composition is not only associated with disease prognosis but also with the risk of surgical complications, e.g. Sarcopenic obesity has been used to predict postoperative pancreatic fistula (POPF) after pancreaticoduodenectomy (PD) (14, 15) and visceral obesity increases the risk of postoperative complications in colon cancer (16, 17). In recent years, body composition parameters have been extensively explored and have been found to affect the prognosis of colorectal cancer patients. SMI and MA have been found to be related to patient prognosis. Dolan, Ross D et al. (18) found that SMI [hazard ratio (HR) 1.50, 95% confidence interval (CI) 1.04–2.18, P = 0.031] was independently associated with overall survival and van Wijk, Laura et al. (19) found that overall survival was lower in patients with both muscle quantity and quality loss compared to other categories. However, relevant studies are still limited, and it is difficult to measure every patient clinically because of the complexity of SMI measurement.

Therefore, in the present study, we propose new clinically easy to measure at CT new predictors of linear skeletal muscle index (LSMI), retrospectively analyzed for oncology-related factors and individual body composition parameters, associated with prognosis in patients with colorectal cancer after radical surgery treatment.

## Materials and Methods

### Study Population

We selected data related to patients admitted to the colorectal cancer surgery ward of the Second Affiliated Hospital of Harbin Medical University from January 2012 to August 2016, including patients who were pathologically diagnosed with colon or rectal cancer and underwent radical surgery at our hospital. We excluded patients with severe systemic disease (eg: severe sepsis, septic shock, hypotension, and multiple organ dysfunction syndrome), other malignancies and those lacking key information (eg: follow-up). Baseline data collected included gender, age, BMI, diagnosis of ileus and diabetes mellitus, body composition parameters, receipt of adjuvant therapy (the standard XELOX, consisting of eight cycles of capecitabine and eight cycles of oxaliplatin) and various tumor characteristics (T and N stage, maximum diameter, presence of metastases, etc.). In this study, the met7astatic location included lung, liver, peritoneum, bone and brain. Based on the previous studies, prognostic factors had been determined (3, 18). All potential prognostic factors were considered as baseline information and calculated after diagnosis and before treatment. As this study was retrospective, we did not obtain relevant information on smoking, alcohol consumption and exercise.

This study was approved by the Ethics Committee of the Second Affiliated Hospital of Harbin Medical University.

### Body Composition and Linear Skeletal Muscle Index Measurements

**Figure 1:**
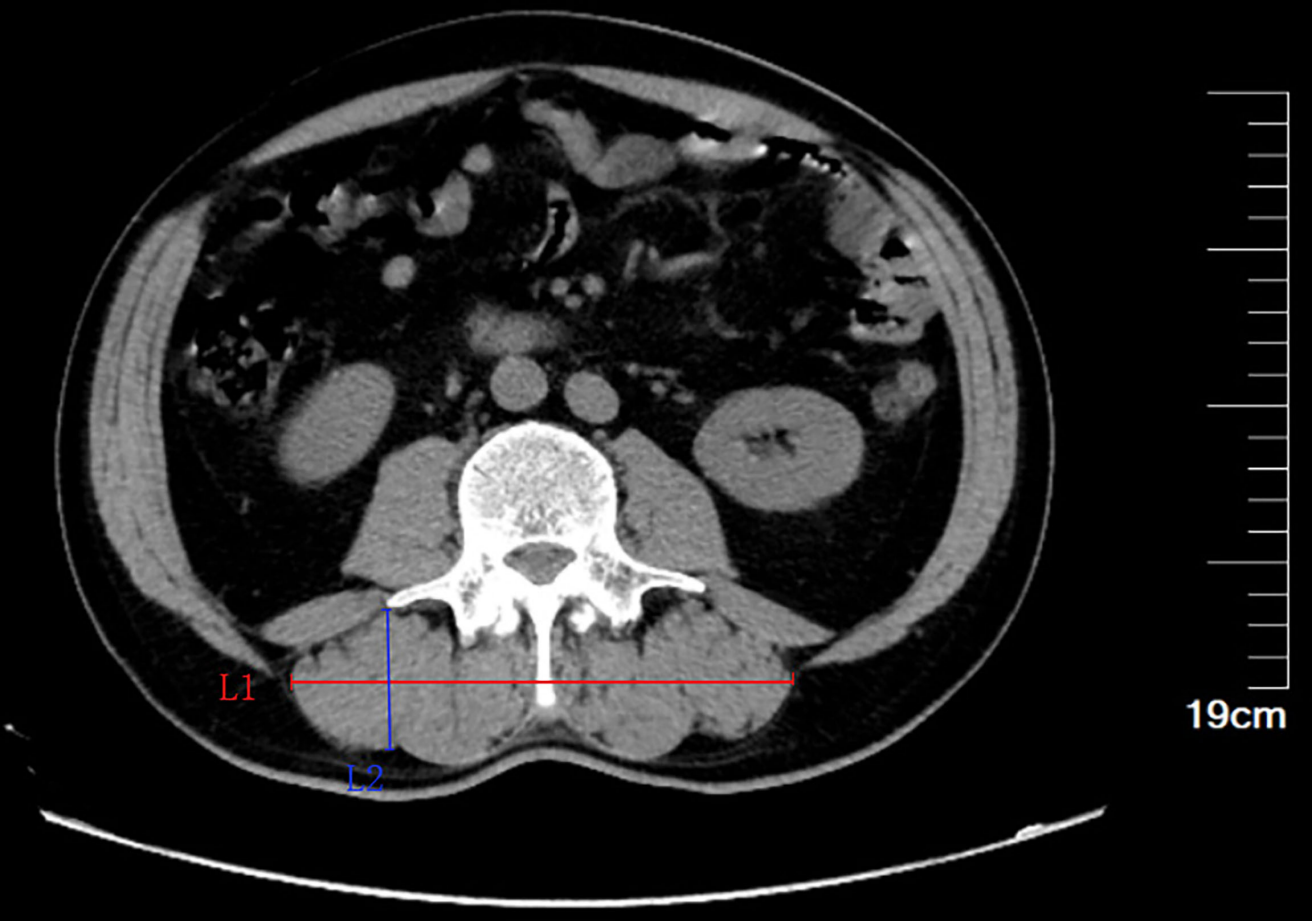
Diagram of transverse and longitudinal diameter of the third lumbar paravertebral muscle group. Ll, maximum diameter in horizontal direction; L2,maximum diameter in vertical direction.

The CT scan results of the 64-slice multi-detector CT scanner (Somatom Definition Flash, Siemens AG, Erlangen, Germany) for each patient were obtained from our center’s database. Volume was used to segment CT data to measure body composition parameters. At the same time, the maximum diameter of paravertebral muscle group in horizontal direction (Ll, cm) and vertical direction (L2, cm) were further measured in the cross section of the third lumbar midpoint. The product of the transverse and longitudinal diameter of the paravertebral muscle group was L1×L2 **(** **Figure 1** **)**. In this study, we defined Ll×L2(cm^2^)/height squared (m^2^) as linear Skeletal muscle index (LSMI).

CT values for muscle tissue were in the range of -29 ~ 150 Hounsfield units; CT values for adipose tissue were in the range of -150 ~ -50 Hounsfield units (20). We measured total psoas muscle area (TPA), total abdominal muscle area (TAMA), subcutaneous fat area (SFA) and visceral fat area (VFA). Posterior renal fat thickness (PPNF) and perirenal fat area (PFA) were measured at the level of the renal vein (21–23), while subcutaneous fat thickness (SCF) was measured at the level of the umbilicus (24, 25). We also measured intermuscular adipose tissue and mean muscle attenuation (MA) at the level of the inferior endplate of L3 (26–28).

### Data Standardization and Outcome Events

We derived highly normalized indices (reported as cm^2^/m^2^) for psoas muscle (PMI) and skeletal muscle (SMI) using the data collected. For example, PMI is total lumbar muscle area (TPA) divided by height squared, and SMI is total abdominal muscle area (TAMA) divided by height squared.

The endpoints of our study were disease-free survival (DFS) and overall survival (OS), defined as all-cause mortality.

### Follow-Up Assessments

Patients were followed up according to NCCN guidelines after radical resection of colorectal cancer (29). The last follow-up was in October 2021. For high-risk patients, physical examinations, CEA and CA19-9, biochemistry, abdominal and pelvic ultrasound were examined every 3 months, and colonoscopy, thoracoabdominal and pelvic CT or MRI were examined every 6 months during the first 2 years after surgery. For other patients, physical examinations, CEA and CA19-9, biochemistry, and abdominal and pelvic ultrasound were reviewed every 3-6 months, and colonoscopy, thoracoabdominal and pelvic CT or MRI were reviewed annually during the first 2 years after surgery. Three to five years after surgery, all patients underwent physical examination, CEA and CA19-9 monitoring, biochemical examination and abdominal and pelvic ultrasound every 6 months, colonoscopy and chest and abdominal and pelvic CT or MRI examinations every year. For all patients, physical examinations, CEA and CA19-9, biochemistry, abdominal pelvic ultrasound, colonoscopy, and thoracoabdominal pelvic CT or MRI were reviewed annually 5 years after surgery.

### Statistical Analysis

Statistical analyses were performed using R version 4.1.2 (R Project for Statistical Computing, Vienna, Austria) and SPSS statistics version 25.0 (IBM, Armonk, NY). X-tile is used to determine the cut-off values. Categorical data was expressed as frequencies (%) and continuous data was expressed as median (min-max). We also used the Kaplan-Meier method to analyze patient survival. In survival analysis, disease-free survival (DFS) and overall survival (OS) were analyzed using standard Cox regression analysis based on the proportional risk assumption. Furthermore, univariate Cox proportional risk models were used to analyze categorical data as well as continuous data, and all univariate significant variables were included in the multivariate Cox proportional risk analysis (p < 0.05).

## Results

### Patient Characteristics

The characteristics of the 1761 post-radical colorectal cancer patients were shown in **Table 1**. Their median age was 62 years (range 23 - 90) and according to the BMI classification, 673 patients were considered as overweight (BMI > 24) and 148 patients were considered as obese (BMI > 28). In these populations, the prevalence of intestinal obstruction, hypertension, diabetes and anaemia were 11.41%, 24.36%, 11.65% and 11.41% respectively. There were slightly more males (n=1082) than females (n=679). All patients had a pathological diagnosis of malignancy, which included 635 patients with T3 and 945 patients with T4 stage **(** **Table 1** **)**.

**Table 1:** Baseline characteristics of study cohort.

	N (%)/Median (Min-Max)
	N (%)
Gender	
Male	1082 (61.44%)
Female	679 (38.56%)
Diabetes mellitus	
Yes	205 (11.64%)
No	1556 (88.36%)
Hypertension (≥130/85mmHg)	
Yes	429 (24.36%)
No	1332 (75.64%)
Anemia	
Yes	346 (19.65%)
No	1415 (80.35%)
Ileus	
Yes	201 (11.41%)
No	1560 (88.59%)
Primary site	
Multiple primary	4 (0.23%)
Right-sided colon	425 (24.13%)
Left-sided colon	436 (24.76%)
Rectum	896 (50.88%)
Tumor size	
>5cm	906 (51.45%)
≥5cm	855 (48.55%)
T stage	
T1	45 (2.56%)
T2	136 (7.72%)
T3	635 (36.06%)
T4	945 (53.66%)
N stage	
N0	1141 (64.79%)
N1	375 (21.29%)
N2	245 (13.92%)
Metastasis	
M0	1637 (92.96%)
M1	124 (7.04%)
Adjuvant therapy	
No	866 (49.18%)
Yes	895 (50.82%)
	Median (Min-Max)
Age	62.00 (23.00-90.00)
BMI	23.05 (12.35-37.03)
MA (Muscle attenuation)	45.41 (2.31-58.90)
IMF (intermuscular)	10.24 (0.90-19.21)
TPA	25.04 (12.20-40.63)
TAMA	129.06 (72.22-196.76)
VFA	105.13 (7.36-186.32)
PFA	21.97 (5.16-30.27)
SFA	108.97 (7.57-209.81)
Abdominal wall fat thickness	2.38 (0.53-4.23)
PPNF	2.15 (0.13-4.23)
VD (visceral fat density)	-91.39 (-115.06-66.72)
SD (subcutaneous fat density)	-92.22 (-118.60-66.61)
PMI	8.93 (3.85-16.62)
LSMI (linear skeletal muscle index)	24.30 (10.02-33.76)
SMI	47.80 (25.82-64.96)

### Survival Analysis

**Figure 2:**
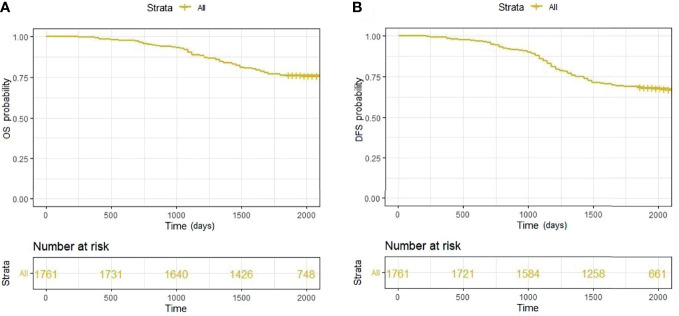
**(A)** Overall survival of all patients; **(B)** Disease-free survival of all patients.

The Kaplan-Meier analysis showed that the 3- and 5-year overall survival rates were 88.87% and 76.38%, respectively **(** **Figure 2A** **)**; the disease-free survival rates were 84.55% and 68.60%, respectively **(** **Figure 2B** **)**. At follow-up, we identified 553 patients who developed resurgence of diseases after radical colorectal cancer surgery and 416 patients who died during the five-year period.

### Prognostic Factors

A total of 27 factors were included in univariate Cox proportional hazard analysis **(** **Table 2** **)**. After that, the significant prognostic factors in univariate COX regression analysis were included in multivariate COX proportional risk analysis. Finally, multivariate COX proportional risk analysis showed that MA, LSMI, SMI, Tumor size, N stage, Metastasis, and postoperative adjuvant therapy were significantly correlated with OS (P < 0.05; **Table 3**). MA, VFA, LSMI, SMI, gender, Tumor size, N stage, Metastasis, and postoperative adjuvant therapy were significantly associated with DFS (P < 0.05; **Table 4**).

**Table 2:** Univariate cox proportional hazard analysis for risk factors of patients’ prognosis.

Characteristics		OS		DFS	
		HR (95%CI)	*P value*	HR (95%CI)	*P value*
Gender	Male	1.0 (ref)	P<0.001	1.0 (ref)	P<0.001
	Female	0.51 (0.41,0.64)		0.59 (0.49,0.71)	
Diabetes mellitus	No	1.0 (ref)	P=0.776	1.0 (ref)	P=0.604
	Yes	0.96 (0.71,1.30)		0.93 (0.72,1.21)	
Hypertension (≥130/85mmHg)	No	1.0 (ref)	P=0.377	1.0 (ref)	P=0.592
	Yes	0.9 (0.72,1.13)		0.95 (0.78,1.15)	
Anemia	No	1.0 (ref)	P=0.565	1.0 (ref)	P=0.638
	Yes	1.07 (0.85,1.36)		1.05 (0.86,1.29)	
Ileus	No	1.0 (ref)	P=0.012	1.0 (ref)	P=0.118
	Yes	1.41 (1.08,1.86)		1.22 (0.95,1.55)	
Primary site	Multiple primary	1.0 (ref)	P=0.296	1.0 (ref)	P=0.265
	Right-sided colon	1.16 (0.16,8.31)		0.68 (0.17,2.73)	
	Left-sided colon	0.99 (0.14,7.06)		0.63 (0.16.2.56)	
	Rectum	0.93 (0.13,6.61)		0.56 (0.14,2.27)	
Tumor size	<5cm	1.0 (ref)	P<0.001	1.0 (ref)	P<0.001
	≥5cm	2.77 (2.25,3.41)		2.09 (1.76,2.47)	
T stage	T1	1.0 (ref)	P<0.001	1.0 (ref)	P<0.001
	T2	1.14 (0.42,3.09)		1.13 (0.56,2.28)	
	T3	2.00 (0.82,4.88)		1.22 (0.65,2.31)	
	T4	2.89 (1.19,6.99)		1.84 (0.98,3.45)	
N stage	N0	1.0 (ref)	P<0.001	1.0 (ref)	P<0.001
	N1	1.89 (1.50,2.37)		2.12 (1.75,2.56)	
	N2	2.76 (2.17,3.50)		2.67 (2.16,3.30)	
Metastasis	M0	1.0 (ref)	P<0.001	1.0 (ref)	P<0.001
	M1	5.57 (4.42,7.03)		6.95 (5.61,8.62)	
Adjuvant therapy	No	1.0 (ref)	P<0.001	1.0 (ref)	P<0.001
	Yes	0.20 (0.16,0.22)		0.40 (0.34,0.48)	
Age		1.00 (0.99,1.01)	P=0.839	1.00 (0.99,1.01)	P=0.592
BMI		0.99 (0.97,1.02)	P=0.665	1.00 (0.97,1.02)	P=0.757
MA (Muscle attenuation)		0.92 (0.91,0.93)	P<0.001	0.94 (0.93,0.94)	P<0.001
IMF (intermuscular)		0.99 (0.97,1.01)	P=0.355	1.00 (0.98,1.02)	P=0.904
TPA		1.00 (0.99,1.01)	P=0.720	1.00 (0.99,1.01)	P=0.755
TAMA		0.93 (0.93,0.94)	P<0.001	0.95 (0.95,0.96)	P<0.001
VFA		1.00 (1.00,1.01)	P=0.054	1.00 (1.00,1.01)	P=0.001
PFA		0.97 (0.95,0.98)	P<0.001	0.98 (0.97,0.99)	P<0.001
SFA		1.00 (1.00,1.01)	P=0.880	1.00 (0.99,1.00)	P=0.962
Abdominal wall fat thickness		1.05 (0.96,1.15)	P=0.268	1.04 (0.96,1.12)	P=0.322
PPNF		1.00 (0.93,1.09)	P=0.922	1.02 (0.95,1.09)	P=0.603
VD (visceral fat density)		1.00 (0.99,1.01)	P=0.924	1.00 (1.00,1.01)	P=0.647
SD (subcutaneous fat density)		1.00 (1.00,1.01)	P=0.609	1.00 (0.99,1.00)	P=0.606
PMI		0.96 (0.92,0.99)	P=0.009	0.99 (0.96,1.02)	P=0.054
LSMI (linear skeletal muscle index)		0.78 (0.76,0.79)	P<0.001	0.82 (0.81,0.83)	P<0.001
SMI		0.80 (0.78,0.81)	P<0.001	0.85 (0.84,0.86)	P<0.001

**Table 3:** Multivariate cox proportional hazard analysis for risk factors of patients’ prognosis (OS).

Characteristics		OS	
		HR (95%CI)	*P value*
Gender	Male	1.0 (ref)	P=0.433
	Female	0.89 (0.68,1.18)	
Ileus	No	1.0 (ref)	P=0.365
	Yes	1.14 (0.86,1.52)	
Tumor size	<5cm	1.0 (ref)	P=0.034
	≥5cm	1.28 (1.02,1.61)	
T stage	T1	1.0 (ref)	P=0.568
	T2	1.61 (0.59,4.42)	
	T3	1.61 (0.65,3.96)	
	T4	1.42 (0.58,3.51)	
N stage	N0	1.0 (ref)	P<0.001
	N1	1.31 (1.03,1.67)	
	N2	1.89 (1.46,2.46)	
Metastasis	M0	1.0 (ref)	P<0.001
	M1	4.55 (3.46,5.97)	
Adjuvant therapy	No	1.0 (ref)	P<0.001
	Yes	0.43 (0.33,0.56)	
MA (Muscle attenuation)		0.96 (0.95,0.97)	P<0.001
TAMA		1.00 (0.99,1.01)	P=0.931
VFA		1.00 (1.00,1.01)	P=0.502
PFA		1.00 (0.98,1.01)	P=0.679
PMI		0.97 (0.94,1.01)	P=0.141
LSMI (linear skeletal muscle index)		0.91 (0.89,0.93)	P<0.001
SMI		0.89 (0.86,0.93)	P<0.001

**Table 4:** Multivariate cox proportional hazard analysis for risk factors of patients’ prognosis (DFS).

Characteristics		DFS	
		HR (95%CI)	*P value*
Gender	Male	1.0 (ref)	P=0.001
	Female	0.67 (0.53,0.84)	
Tumor size	<5cm	1.0 (ref)	P=0.020
	≥5cm	1.25 (1.04,1.51)	
T stage	T1	1.0 (ref)	P=0.080
	T2	1.63 (0.80,3.31)	
	T3	1.13 (0.54,1.95)	
	T4	1.11 (0.53,1.93)	
N stage	N0	1.0 (ref)	P<0.001
	N1	1.43 (1.16,1.75)	
	N2	1.64 (1.31,2.06)	
Metastasis	M0	1.0 (ref)	P<0.001
	M1	4.04 (3.19,5.12)	
Adjuvant therapy	No	1.0 (ref)	P=0.033
	Yes	0.80 (0.65,0.98)	
MA (Muscle attenuation)		0.97 (0.97,0.98)	P<0.001
TAMA		1.00 (0.99,1.01)	P=0.828
VFA		1.01 (1.00,1.01)	P<0.001
PFA		1.00 (0.99,1.01)	P=0.902
PMI		1.00 (0.96,1.03)	P=0.801
LSMI (linear skeletal muscle index)		0.93 (0.91,0.95)	P<0.001
SMI		0.93 (0.90,0.96)	P<0.001

We further found that patients with higher MA, LSMI and SMI values had a lower risk of all-cause mortality, while patients with tumor size ≥5cm, higher N stage, presence of distant metastases and no postoperative adjuvant chemotherapy had a higher risk of all-cause mortality. And patients with higher MA, LSMI and SMI values had a lower risk of tumor progression, while patients with higher VFA, male patients, tumor size ≥5 cm, higher N stage, presence of distant metastases and no postoperative adjuvant chemotherapy had a higher risk of tumor progression.

### Cut-Off Values for Prognostic Factors

**Figure 3:**
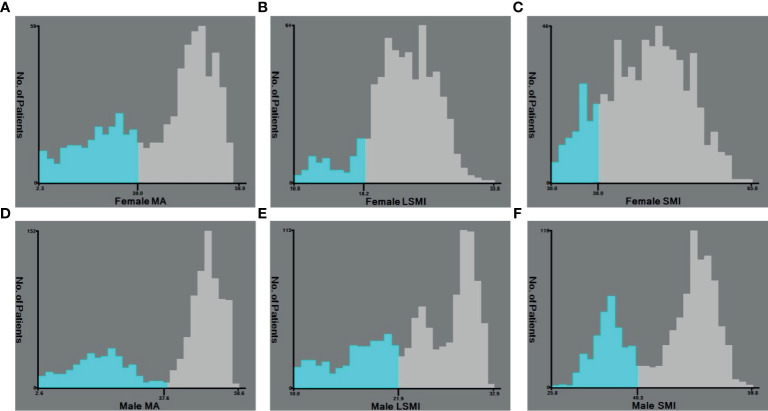
**(A)** Cut-off values of Female MA; **(B)** Cut-off values of Female LSMI; **(C)** Cut-off values of Female SMI; **(D)** Cut-off values of Male MA; **(E)** Cut-off values of Male LSMI; **(F)** Cut-off values of Male MA.

Based on the overall survival (OS), we found that female patients with MA <30.0HU (**Figure 3A**), LSMI <18.2 (**Figure 3B**), and SMI <38.0 (**Figure 3C**) had a poorer prognosis; male patients with MA <37.6HU (**Figure 3D**), LSMI <21.9 (**Figure 3E**), and SMI <40.3 (**Figure 3F**), male patients had a worse prognosis.

### Nomograms Construction and Clinical Performance

**Figure 4:**
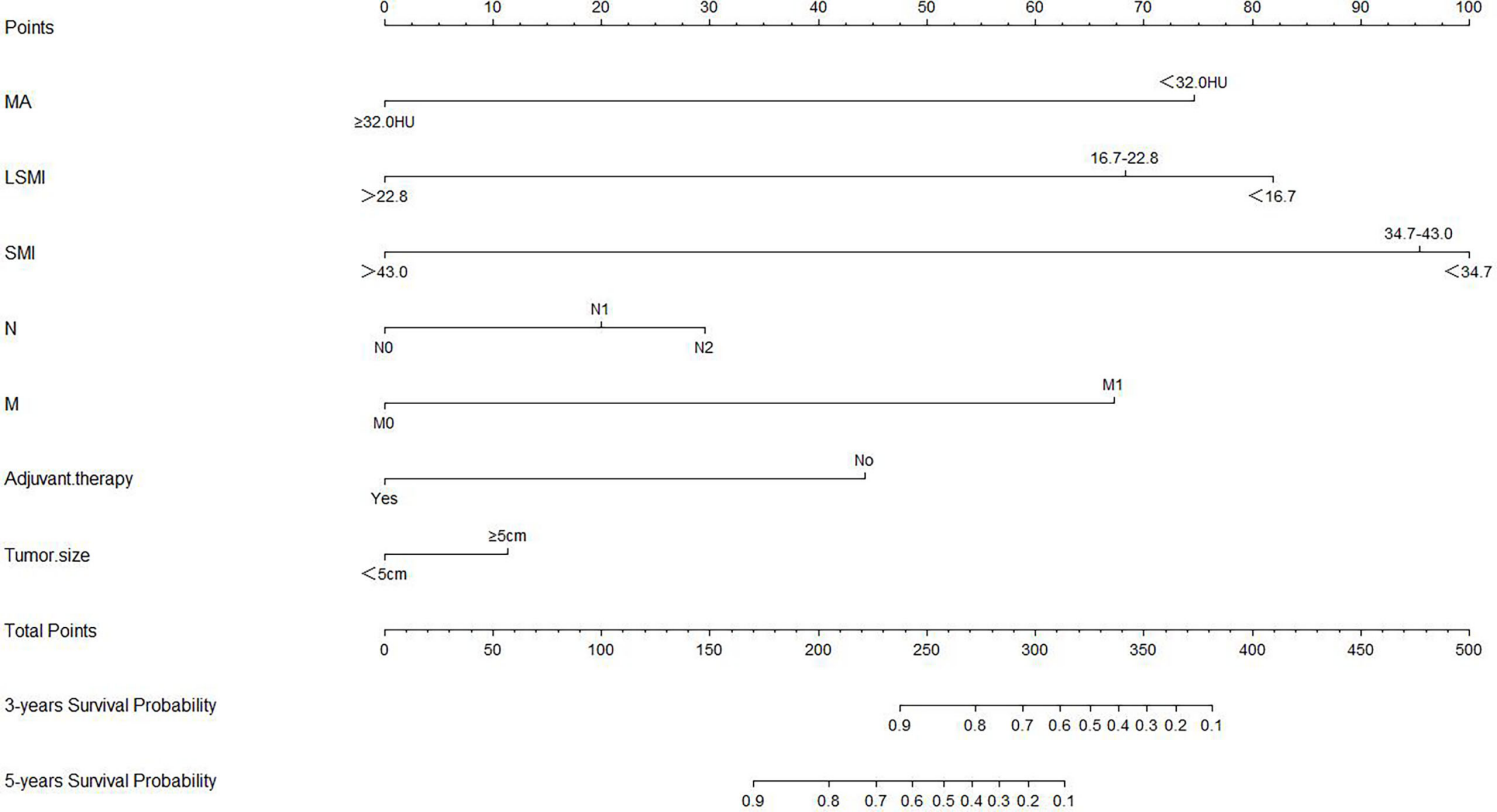
Nomogram for predicting overall survival in colorectal cancer patients.

**Figure 5:**
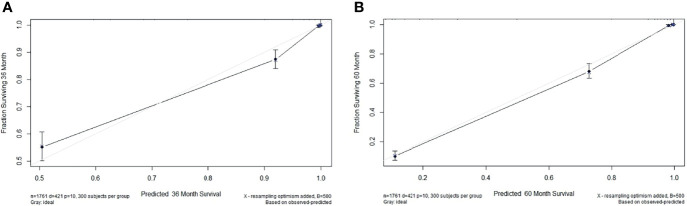
**(A)** The calibration plots of the nomogram for 3-year overall survival. **(B)** The calibration plots of the nomogram for 5-year overall survival.

**Figure 6:**
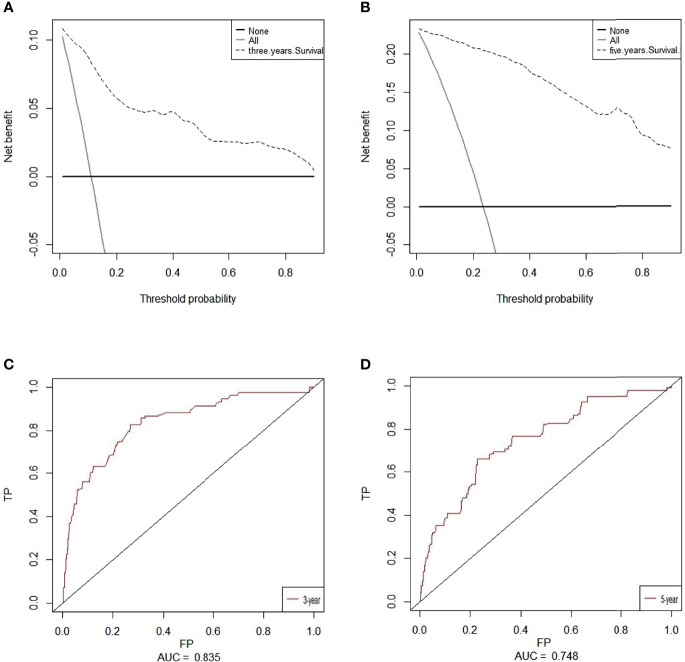
Decision curve analysis (DCA) of the nomogram of based on 3-year **(A)** and 5-year **(B)** overall survival. The x-axis and the y-axis were the threshold probability and the net benefit, respectively. Receiver operating characteristic (ROC) analyses of the nomogram based on 3-year **(C)** and 5-year **(D)** overall survival.

Based on overall survival, we constructed a nomogram using seven independent predictors including MA, LSMI, SMI, tumor size, N stage, metastasis and adjuvant therapy after multivariate COX regression analysis **(** **Figure 4** **)**. The calibration curves at 3 and 5 years were close to 45 degrees **(** **Figure 5** **)**, indicating that the nomogram has good calibration ability. DCA curves at 3 and 5 years showed that the model had good clinical performance **(** **Figure 6** **)**. The ROC was used in this study to determine the predictive effect of the model. The 3-year and 5-year AUC values for this nomogram were 0.835 (95% CI, 0.818 - 0.852) and 0.748 (95% CI, 0.733 - 0.763) **(** **Figure 6** **)**.

## Discussion

In patients with colorectal cancer, quantitative calculation of skeletal muscle area by abdominal CT images is the recognized gold standard for the diagnosis of sarcopenia (30). However, because this method requires a lot of time for each patient to use software to perform manual segmentation, it is currently not clinically conditions to diagnose sarcopenia for each patient (31). This study is the first to find that the product of the transverse and longitudinal diameter of the paraspinal muscle group/square of the height (Linear Skeletal muscle index) on abdominal CT images of the third lumbar spine can be used as a new prognostic factor for colorectal cancer. This method has the advantages of saving time and being easy to measure. When LSMI <18.2 in female patients, and LSMI <21.9 in male patients, the prognosis of patients is worse.

At the same time, we found that few studies have examined muscle and fat tissues among Chinese people. To our knowledge, this is the first time that a combination of muscle and adipose-related parameters, linear Skeletal Muscle Index (LSMI), and oncological parameters has been used to determine survival outcomes in CRC patients undergoing radical surgery. We found that skeletal muscle mass index (SMI), muscle attenuation (MA), linear skeletal muscle index (LSMI), tumor size, N stage, metastasis, and postoperative adjuvant therapy were significantly correlated with the survival rate of colorectal cancer patients after radical resection.

Skeletal muscle is the largest organ in non-obese individuals and it accounts for 40% of body mass (32). Muscle and fat are two important components of the body and a reliable method of assessing the amount and distribution of muscle and fat in the body is clinical imaging and CT scan analysis at the level of the third lumbar vertebra is considered to be the gold standard for measuring body composition parameters. There may be some amount of skeletal muscle loss, either during cancer radiotherapy or malignancy development (33–35), and loss of muscle mass or quality may shorten overall survival (36). The amount and distribution of adipose tissue affects clinical outcomes differently in different types of tumors (10, 11).

SMI is used to measure body muscle mass and is often used as a standard indicator to diagnose sarcopenia, while MA is used to measure muscle quality (37). Skeletal muscle is the largest protein storage site in the body, and it secretes hundreds of myostatin peptides that affect immune function, adipose tissue oxidation, insulin sensitivity and systemic metabolism. Studies have shown that sarcopenia can be mediated through mechanisms such as autophagy (38), disturbances in adipose tissue metabolism (39), oxidative stress (40) and systemic inflammation (41). Loss of MA and SMI is associated with poor prognosis in patients with a variety of solid tumors, such as bladder cancer, lung cancer, breast cancer and gastric cancer (42–45).

Low MA is associated with physical inactivity, obesity and muscle atrophy, which often results in metabolic disturbances, severe postoperative complications and a systemic inflammatory response (46). Inflammation and oxidative stress activate the ubiquitin-proteasome system and apoptosis-inducing proteins, and inhibit insulin-like growth factors (47). Studies have found that skeletal muscle attenuation density is negatively correlated with muscle fibre fat content, and muscle mass and strength are positively correlated (48).

Research demonstrating that muscle mass and quantity are associated with mortality could be used as a framework to test the hypothesis that targeted interventions for muscle status may provide clinical benefit in this population. In this framework, measures of muscle mass and quantity could be used as therapeutic targets (i.e. biomarkers) to guide the development of early treatment. For example, resistance training combined with exercise, nutrition and medication might improve muscle status.

Most of the pathological types of colorectal cancer are adenocarcinoma, which contains a large number of lipid droplets. Therefore, lipid metabolism may play an important role in colorectal cancer. Adipose tissue is an endocrine organ with high metabolic activity, with visceral adipose tissue (VAT) being the most metabolically active (49). Environmental and genetic factors lead to abnormal fat distribution in the body. The accumulation of VAT does not necessarily correlate positively with BMI and patients with low BMI may have higher than normal levels of visceral adipose tissue. Visceral adipose tissue releases adipokines such as IL-6, TNF-a, VEGF, and fibrinogen activator inhibitor-1 that are involved in inflammation and angiogenesis, while decreasing lipocalin expression (50). Meanwhile, many studies have reported that reduced serum lipocalin levels are associated with an increased incidence of common malignancies, including breast, colon and prostate cancers (51, 52). Visceral obesity is one of the manifestations of abnormal body fat distribution under the influence of multiple genetic and environmental factors.

Visceral obesity increases the burden of endoplasmic reticulum, triggers an endoplasmic reticulum stress response and impairs insulin signaling pathways. These factors lead to chronic inflammatory response and insulin resistance, magnifying surgical stress and aggravating surgical complications (17). Polyunsaturated fatty acids (PUFA) have also been found to intervene in the development and progression of colorectal cancer (53). Meanwhile, our results also suggest that visceral fat area (VFA), a fat parameter measured in the third lumbar spine, may significantly influence the invasion and development of colorectal cancer.

The current study has some limitations, and we need to acknowledge that the present study did not retrospectively obtain data on important diagnostic factors related to sarcopenia, such as walking speed and patient self-report on the SARC-F questionnaire (54). Additionally, we did not obtain parameters related to tumor nutrition, such as grip strength, triceps skinfold thickness and the PG-SGA score patient self-assessment scale (55), because some body composition parameters are directly affected by the patient’s nutritional status. In addition, the relatively small cohort and retrospective nature of data collection are potential sources of bias. Future research should focus on validating and refining these results.

Besides, we consider providing some commentary on the next steps for this line of research. In the future, we plan to examine these indicators compared to sarcopenia or other independent prognostic factors for colorectal cancer and explore whether imaging is the most cost-effective tool in this setting. Moreover, we will determine if there are ways to assess proxies for skeletal muscles (e.g., exercise behavior, nutrition, walking pace) in the real-world.

## Conclusion

We demonstrate for the first time that linear skeletal muscle index is an independent and powerful prognostic factor for patients with colorectal cancer, and furthermore we find that more refined body composition parameters than BMI combined with oncology-related parameters may provide a more comprehensive assessment of patient prognosis. Our results may provide a reference in their postoperative management.

## Data Availability Statement

The original contributions presented in the study are included in the article/supplementary material. Further inquiries can be directed to the corresponding author.

## Ethics Statement

This study was reviewed and approved by the Research Ethics Committee of the Second Affiliated Hospital of Harbin Medical University, China. Patients/participants provided written informed consent to participate in this study.

## Author Contributions

YW: conceptualization, data capture, investigation, writing-original, formal analysis. YLMW and HZ: formal analysis, software, validation and writing-review and editing. LA: software, visualization and data capture. ZW and XJ: data collection, data management and software. YL: formal analysis and investigation. GY: visualization and data capture. HX: writing-review and editing. GL: software, investigation, supervision and writing-revision. CW: investigation and methodology. GW: supervision, validation and project management. ML: supervision, project management, funding acquisition. All authors of this article made significant contributions and approved the submitted version.

## Funding

This project was supported by the Natural Science Foundation of Heilongjiang Province, China - Joint Guidance Project (Grant No. LH2020H066).

## Conflict of Interest

The authors declare that the research was conducted in the absence of any commercial or financial relationships that could be construed as a potential conflict of interest.

## Publisher’s Note

All claims expressed in this article are solely those of the authors and do not necessarily represent those of their affiliated organizations, or those of the publisher, the editors and the reviewers. Any product that may be evaluated in this article, or claim that may be made by its manufacturer, is not guaranteed or endorsed by the publisher.
